# The effect of basic psychological needs in physical education on exercise behavior in young adulthood: the mediating role of self-efficacy and moderated role of gender

**DOI:** 10.3389/fpsyg.2026.1757499

**Published:** 2026-02-24

**Authors:** Xuening Li, Donghai Wang, Weijun Xie, Jing Wang

**Affiliations:** 1 Department of Physical Education, Nanjing University of Posts and Telecommunications, Nanjing, China; 2 College of Physical Education, Dalian University, Dalian, China

**Keywords:** basic psychological needs, exercise behavior, gender, physical education, self-efficacy, young adulthood

## Abstract

**Background:**

Physical inactivity during young adulthood has become a global public health concern. Based on basic psychological needs theory, satisfying these needs in physical education may be positively associated with exercise behaviors; however, the underlying mechanisms and gender differences remain insufficiently explored.

**Methods:**

A cross-sectional survey was conducted with 3,188 students aged 18 to 21 years old (Mean = 19.26, SD = 0.93; 47.55% males). Participants completed questionnaires measuring basic psychological needs, self-efficacy, and exercise behavior. Structural equation modeling (SEM) tested the mediating role of self-efficacy and gender differences via multi-group analysis.

**Results:**

Findings showed that males scored significantly higher than females in competence, autonomy, and relatedness needs, as well as self-efficacy and exercise behavior, compared with females. All gender-related associations reached statistical significance. SEM results showed that the three needs significantly and directly associated with exercise behavior, with self-efficacy serving as a parallel mediator in this relationship. When gender was included in the model, multi-group SEM indicated significant gender differences across all three pathways. Specifically, the association of competence need on exercise behavior was weaker among female college students, whereas the association of autonomy need was stronger. Moreover, competence need was more strongly associated with self-efficacy among male students.

**Conclusion:**

Satisfaction of these needs correlates with higher exercise behavior, and this association is mediated by enhanced self-efficacy. Further intervention studies should account for gender differences to optimize their outcomes.

## 1 Introduction

The health challenges facing today’s college students have grown increasingly severe. According to the eighth national survey on student physical health (Department of Physical Education, Health and Art Education, Ministry of Education, 2021), the downward trend in physical fitness among college students has yet to be reversed. For college students, sedentary study habits, prolonged screen time, and late-night gaming have become increasingly prevalent, substantially reducing the time available for physical activity (Meghji et al., 2025). At the same time, the mass expansion of higher education and intense competition in the job market have plunged many students into “academic-career” anxiety (Li, 2012), further reinforcing a sedentary, low-activity lifestyle. As a result, not only is the frequency of student exercise insufficient, but also the intensity and effectiveness of their workouts are also compromised (Chen, 2024). In this context, promoting and strengthening regular physical activity among college students is more important than ever. Previous research showed that basic psychological needs in PE significantly bolster students’ exercise intentions, the underlying mechanism exhibits substantial interindividual variability (Li et al., 2024). In PE contexts, basic psychological needs may enhance exercise self-efficacy, thereby facilitating both initiation and persistence in physical activity. Moreover, gender differences in physical activity patterns are well documented. Whether such gender differences moderate the pathway from class-based psychological needs through self-efficacy to exercise behavior remains an open empirical question. Thus, the present study uses structural equation modeling (SEM) to test the relationship “basic psychological needs in PE → self-efficacy → exercise behavior” and to examine gender as a moderator of these relationships.

## 2 Literature review

Self-Determination Theory (SDT) posits that humans are innately endowed with three fundamental psychological needs: autonomy, competence, and relatedness. The fulfillment of these needs is essential for individuals’ growth, healthy development, and overall well-being (Deci and Ryan, 2000). In the context of PE, the need for autonomy refers to an individual’s sense of volition and freedom of choice in sports-related behaviors (Vlachopoulos et al., 2011). The need for competence reflects an individual’s perception of efficacy, derived from skill acquisition and task accomplishment during interactions within the PE environment (Li et al., 2024). The need for relatedness emphasizes the emotional dimension of teacher-student interactions and peer collaboration, encompassing psychological requirements for understanding, respect, and support, thereby fostering a sense of belonging in the classroom (Ruzek et al., 2016). When these needs are satisfied, individuals acquire the psychological resources necessary to experience more self-determined and integrated forms of motivation (Ryan and Deci, 2000). High-quality exercise behaviors occur when individuals engage in physical activity through integrated forms of motivation, characterized by internalized identification with and genuine valuing of the outcomes of participation (Teixeira et al., 2012).

Interestingly, prior research has established the positive association of basic psychological needs in PE on exercise behavior, but most discussions have emphasized the overall, macro-level role of psychological needs (Sylvester et al., 2018). Although SDT theory holds that autonomy, competence, and relatedness are equally vital to psychosocial functioning (Ryan and Deci, 2000), this assumption often overlooks the dynamic and context-dependent nature of need prioritization in real-world settings. For instance, Vlachopoulos et al. (2013) found that the relative salience of the three psychological needs varies across cultural contexts and environmental conditions. Similarly, empirical research on Chinese athletes show that competence and autonomy are significantly associated with sports participation persistence, which may stem from their competitive performance-oriented training context (Zhang and Miao, 2024). While these findings are relevant to college students due to their shared engagement in structured physical activities, the focus of college physical education on cultivating lifelong exercise habits and fostering interpersonal collaboration (Huan, 2024) implies potential differences in the prioritization of psychological needs. College PE courses incorporated team-based sports and collaborative activities that emphasize interpersonal interaction (Zhang, 2023). In such contexts, relatedness needs may play a more prominent role than in elite athletic training. Existing research on the hierarchical structure of these basic psychological needs in college PE remains limited, highlighting the necessity of the current study to explore the association of PE-based psychological needs on young adults’ exercise behavior.

Self-efficacy, defined as an individual’s confidence in their ability to successfully complete specific activities (Bandura, 1993), is a key cognitive determinant of exercise behavior (Sweet et al., 2012). Higher levels of self-efficacy correlate with greater enthusiasm for physical activity, reflected in increased frequency, intensity, and duration (Samson and Solmon, 2011), and confidence in motor abilities is crucial for exercise engagement (Zhao et al., 2024). Within the framework of SDT, the satisfaction of basic psychological needs is a critical precursor to enhanced self-efficacy, with consistent positive associations between the two (Li et al., 2024). Notably, competence need and self-efficacy share conceptual overlap, as both emphasizing beliefs in one’s ability to act autonomously and meet expectations (Rodgers et al., 2014). It should be clarified, however, that their core connotations and roles are inherently distinct: competence need is an innate psychological need for mastery experiences (Ryan and Deci, 2000), while self-efficacy is a context-specific belief in one’s capacity to achieve goals (Bandura, 1977). This distinction clarifies their roles in the model: the satisfaction of competence need is positively associated with self-efficacy, which mediates the association between need satisfactions on exercise behavior. For example, targeted technical guidance supporting competence need enhances individuals’ confidence in completing physical activities (Ümmet, 2017). Similarly, Sweet et al. (2012) reported that autonomy and relatedness needs are positively associated with self-efficacy. Few studies have explored this relationship in PE, but PE’s salient performance feedback provides a unique context for this mechanism. Thus, self-efficacy may mediate the link between basic psychological needs in PE and college students’ exercise behavior.

Interestingly, the extent to which individual’s psychological needs are satisfied and their function efficacy may also be associated with by gender (Manzano-Sánchez et al., 2023), although consensus is not reached. Fraguela-Vale et al. (2020) found male college students generally reported higher levels of basic psychological needs in PE than females, with the strongest gender difference observed in competence need. Given the observed associations, the link between competence, self-efficacy, and exercise behavior may differ across gender groups. Similarly, Rijo et al. (2014) documented gender differences in autonomy need, suggesting that the association between autonomy need and exercise behavior may differ across genders. Manzano-Sánchez et al. (2023) noted balanced support for psychological need in PE mitigates gender disparities, reinforcing gender’s moderating potential and hinting at contextual boundaries. Additionally, other research confirmed male college students exhibit higher self-efficacy than females (Fraguela-Vale et al., 2020). As self-efficacy mediates basic psychological needs and exercise behavior, this gender gap suggests the mediating pathway may operate differently across genders. Collectively, these findings explicitly justify our core propositions: (1) gender moderates the direct relationship of basic psychological needs on exercise behavior relationship; (2) gender moderates the indirect relationship of basic psychological needs on exercise behavior via self-efficacy. These findings underscore the possibility that gender may serve as a moderating factor in the relationship between basic psychological needs, exercise behavior, and self-efficacy among college students.

In summary, although prior research has preliminarily explored the associative mechanisms linking basic psychological needs, self-efficacy, and exercise behavior in PE, it has not yet examined the importance and experiences of competence, autonomy, and relatedness independently. In particular, gender difference may moderate the associations among these variables. This current study thus aims to address three core questions: (1) How do the needs of competence, autonomy, and relatedness in PE relate to college students’ exercise behavior, and what is the relative importance of these three needs in this association? (2) Does self-efficacy serve as a mediating role in the association between competence, autonomy, and relatedness needs and exercise behavior? (3) Does gender moderate the associations between basic psychological needs, self-efficacy, and exercise behavior?

## 3 Method

### 3.1 Study design

A cross-sectional survey design was adopted in this study to explore the relationships among basic psychological needs in PE, self-efficacy, gender, and exercise behavior among college students. All data were collected through standardized questionnaires to ensure the consistency and objectivity of measurement. The questionnaires were distributed and collected on-site in schools, and trained researchers provided unified guidance to participants to reduce potential response biases.

### 3.2 Participants

We employed a simplified cluster sampling approach to randomly recruit participants from five universities across Shandong, Hebei, Henan, Jiangsu, and Fujian provinces, China between March 27 and April 11, 2025. The recruited students were freshmen, and all participants completed the questionnaire anonymously via the “Wenjuanxing” platform in China. A total of 3,225 students were initially surveyed and ranged in age from 18 to 21 years old. After excluding 37 cases due to patterned responses or faulty data, the final sample comprised 3,188 students, yielding a valid response rate of 98.85%. There were 1,516 boys (47.55%) and 1,672 girls (52.45%), with a mean age of 19.26 ± 0.93 years. The study was approved by University Academic Ethics and Integrity Committee (2025B002), and conducted in accordance with the Declaration of Helsinki.

### 3.3 Measure

The Basic Psychological Needs in Exercise Scale developed by Vlachopoulos and Michailidou (2006) was used to asses students’ satisfaction to evaluate the extent of satisfaction of psychological needs. The scale consists of 12 items and divided into three dimensions, competence, autonomy, and relatedness. Each item was assessed on a 7-point Likert scale (1 = “strongly disagree,” 7 = “strongly agree”), with higher scores indicating higher levels of need satisfaction. In this study, we used a Chinese edition of the BPNES, which has been translated and validated (Liu et al., 2013). The overall Cronbach’s alpha coefficient for was 0.925 in the present study, indicating high internal consistency reliability; the Cronbach’s alpha coefficient for competence, autonomy, and relatedness were 0.882, 0.832, and 0.881, respectively. Additionally, a confirmatory factor analysis (CFA) revealed a model fit (*χ*^2^/df = 6.874, GFI = 0.967, NFI = 0.975, IFI = 0.977, CFI = 0.977, and RMSEA = 0.071).

Exercise behavior was measured using the Physical Exercise Questionnaire revised by Wu (2016). The author of the questionnaire has made it publicly available and free for use. There are eight items in the questionnaire for assessing exercise commitment (“I often participate in sports activities”) and exercise adherence (“I find it difficult to accept a lifestyle that lacks physical exercise”), each subscale with 4 items. Each item had to be rated on a 5-point Likert scale, ranging from strongly 1 (strongly disagree) to 5 (strongly agree). The higher the scores represent greater positivity and persistence in exercise behavior. In the present study, the Cronbach’s alpha coefficient for the scale was 0.916, with Cronbach’s alpha coefficient for each subscale being 0.880 and 0.870, respectively. The model fit was good (*χ*^2^/df = 5.640, GFI = 0.996, NFI = 0.997, IFI = 0.998, CFI = 0.998, RMSEA = 0.038).

To measure individual’s the level of self-efficacy, we used a Chinese version of General Self-Efficacy Scale, which has been widely validated and demonstrates good reliability in Chinese populations (Zhang and Schwarzer, 1995). Each item scored using a 4-point Likert scale (1 = “completely disagree,” 4 = “completely agree”), with higher scores indicating higher levels of self-efficacy. In the present study, the Cronbach’s alpha coefficient for this scale was 0.948. The CFA result reveals a good fit (*χ*^2^/df = 5.771, GFI = 0.968, NFI = 0.933, IFI = 0.981, CFI = 0.981, RMSEA = 0.077).

### 3.4 Data analysis

The data analysis in this study was mainly divided into three steps. First, Excel was used for the preprocessing of collected data. Second, SPSS 27.0 was employed to conduct t-tests, descriptive statistics, and correlational analyses on the collected data. Finally, SEM was constructed using Amos 24.0 to investigate the direct and indirect associations between basic psychological needs and exercise behavior. Goodness-of-fit indices were used to evaluate the fitness of the mediation model (Bollen, 1989; Kim and Faith, 2020). Specifically, the chi-square (*χ*^2^) value was used to assess the degree of fit between the proposed model and the data, with a *p* > 0.05 indicating a good model fit. The root mean square error of approximation (RMSEA) was applied to evaluate whether the model supported the factor structure. A value less than 0.05 represents an excellent fit, while a value between 0.05 and 0.08 indicates an acceptable fit. The comparative fit index (CFI), normed fit index (NFI), goodness-of-fit index (GFI), and adjusted goodness-of-fit index (AGFI) were also used as important measurement indices, and values higher than 0.90 for each index were regarded as an acceptable model fit. In addition, the bootstrap analysis with 5,000 random resamples was performed to test the significance of the indirect pathways and estimate the 95% bias-corrected confidence intervals. The accepted level of significance was *p* < 0.05. Additionally, multigroup CFA tested measurement invariance across gender was tested following the sequential procedure for equivalent factor structure, weight invariance and, structural weight invariance (Vandenberg and Lance, 2000). The change in ΔCFI was adopted as the criterion for invariance evaluation, with a cutoff of |ΔCFI| ≤ 0.01 indicating that the invariance hypothesis was supported (Rutkowski and Svetina, 2014). It is worth noting that participants were recruited from multiple universities, resulting in a nested data structure with students clustered within institutions. Thus, we adopted intra-cluster correlation coefficient (ICC) for the primary variables to assess the potential impact of clustering using two-level random-intercept null models, with students nested within universities, but multilevel modeling was not applied in the present study (Eldridge et al., 2009). This decision was based on the study’s primary focus on individual-level psychological mechanisms and the relatively limited number of higher-level clusters, which may reduce the stability and interpretability of multilevel estimates. Therefore, all analyses were conducted at the individual level, and the potential impact of clustering is acknowledged.

## 4 Results

### 4.1 Common method variance test

To mitigate concerns regarding common method variance (CMV) stemming from self-reported data (Podsakoff et al., 2003), we performed statistical analyses to assess the potential influence of CMV on the validity of study results. Firstly, we adopted the Harman’s single-factor test. The results showed that the first factor explained 35.07% of the total variance, well below the critical threshold for CMV dominance. Additionally, we incorporated a latent common method factor into the Partial Least Squares (PLS) model—with indicators encompassing all principal constructs’ indicator (Podsakoff et al., 2003; Williams et al., 2003). This approach controls for systematic item-level variance that is independent of the covariance attributable to the constructs of interest (Liang et al., 2007). As shown in Supplementary Table S1, the results indicate that the average substantively explained by the indicators was 0.549, whereas the average method-related variance was 0.055, yielding an approximate ratio of 10:1. Moreover, most method factor loadings were non-significant. Given the small magnitude and insignificance of method variance, we suggest that CMV does not pose a substantial threat to the validity of the present study’s findings.

### 4.2 Analysis of differences in basic psychological needs in PE, self-efficacy, and exercise behavior among different gender groups

As shown in Table 1, the results of t-test analysis showed that the mean levels of autonomy, competence, relatedness, self-efficacy, and exercise behavior were higher for male college students than female (*p* < 0.001).

**Table 1:** Gender differences in autonomy, competence, relatedness, self-efficacy and exercise behavior.

Variables	Male (*n* = 1,516)	Female (*n* = 1,672)	F	*t*	*p*
Competence	19.25 ± 5.17	18.27 ± 5.05	1.52	5.42	<0.001
Autonomy	20.94 ± 4.64	19.96 ± 4.61	0.50	6.00	<0.001
Relatedness	21.29 ± 4.72	20.72 ± 4.55	0.09	3.51	<0.001
Exercise behavior	26.12 ± 6.68	22.56 ± 5.65	51.71	16.19	<0.001
Self-efficacy	26.38 ± 6.63	25.73 ± 6.31	1.31	2.85	0.004

### 4.3 Descriptive statistics and preliminary analyses

Table 2 presents descriptive statistics and correlations for the study variables. Pearson correlation analysis revealed that, with exception of age, all variables were significant intercorrelated, with coefficients ranging between *r* = 0.05 and *r* = 0.70. These findings provided theoretical basis for the subsequent investigation of the mediating role of self-efficacy in the relationship autonomy, competence, relatedness and on exercise behavior.

**Table 2:** Descriptive statistics and Pearson correlation coefficient among research variables (*N* = 3,188).

Variables	M ± SD	1	2	3	4	5	6	7
1. Sex	—	1	—	—	—	—	—	—
2. Age		—	1	—	—	—	—	—
3. Self-efficacy	26.04 ± 6.47	−0.05**	0.014	1	—	—	—	—
4. Competence	18.73 ± 5.13	−0.10**	−0.033	0.51**	1	—	—	—
5. Autonomy	20.42 ± 4.65	−0.11**	−0.030	0.45**	0.68**	1	—	—
6. Relatedness	20.99 ± 4.64	−0.06**	−0.015	0.49**	0.60**	0.70**	1	—
7. Exercise behavior	24.25 ± 6.41	−0.28**	0.026	0.32**	0.53**	0.41**	0.31**	1

*p < 0.05, **p < 0.01.

### 4.4 Discriminant validity between competence need and self-efficacy

Given the conceptual proximity between competence need and self-efficacy, additional analyses were conducted to ensure adequate discriminant validity and to rule out potential construct redundancy. Based on a unified two-factor measurement model, discriminant validity was assessed using the criteria proposed by Fornell and Larcker (1981). The results indicated that the average variance extracted (AVE) values for competence need and self-efficacy were 0.81 and 0.85, respectively, both well above the recommended threshold of 0.50. Moreover, the square roots of the AVE values for competence need (0.90) and self-efficacy (0.92) were all greater than the inter-construct correlation coefficient (*r* = 0.56, *p* < 0.001). CFA further showed that all indicators loaded significantly onto their respective constructs, with standardized loadings ranging from 0.73 to 0.92 and composite reliability (CR) values exceeding 0.70, indicating satisfactory internal consistency (Hair et al., 2009). The unified two-factor model exhibited excellent model fit (*χ*^2^/df = 2.9, RMSEA = 0.053, CFI = 0.954, NFI = 0.952, IFI = 0.954). Although the fit indices were slightly lower than those of the separate single-factor models, the differences were negligible (<0.02), and overall fit remained within the excellent range. Within the unified model, the latent correlation between competence need and self-efficacy was moderate (*r* = 0.56, 95% CI [0.49, 0.63]), consistent with their theoretical distinction and well below the threshold indicative of construct redundancy. Collectively, these findings provide strong methodological support for modeling competence need and self-efficacy as independent constructs in subsequent hypothesis testing.

### 4.5 Mediation analysis

The ICC values for the exercise behavior, basic psychological needs and self-efficacy were small (all <0.01), indicating minimal between-university variance. Therefore, the clustered data structure was unlikely to substantially bias parameter estimates, and individual-level analyses were deemed appropriate.

**Figure 1:**
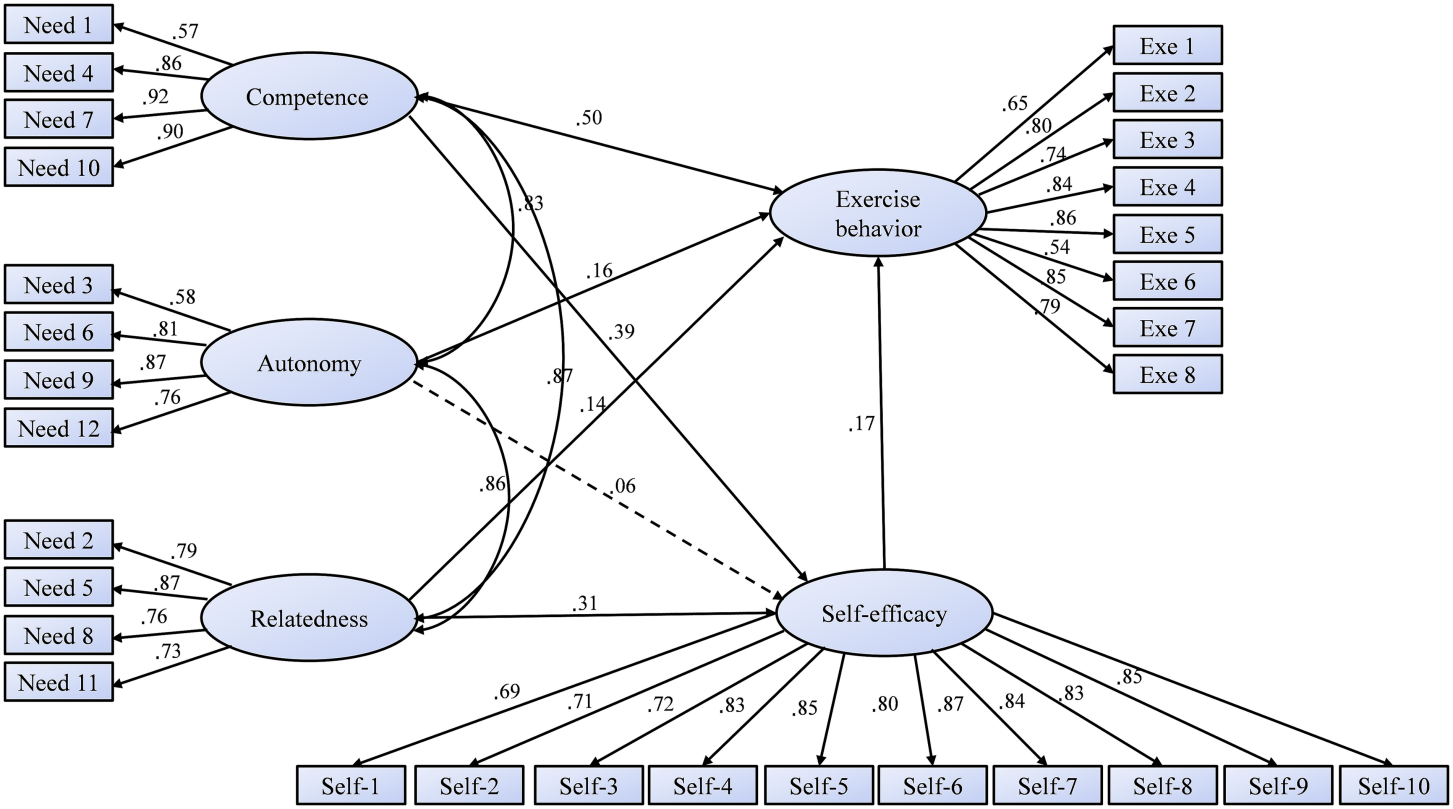
The structural equation modeling of autonomy, competence, relatedness, self-efficacy, and exercise behavior.

Based on theoretical hypotheses, to further explore the relationships between students’ basic psychological needs for autonomy, competence, and relatedness, self-efficacy, and exercise behavior, an AMOS-SEM approach was used to conduct path analysis. Age and gender were controlled for as covariates. The fit indices extremely well-fitting model: *χ*^2^/df = 5.701, RMSEA = 0.058, GFI = 0.901, NFI = 0.938, IFI = 0.943, and CFI = 0.943. All estimated direct paths are presented in Figure 1. Exercise behavior showed a significant positive relationship with competence (*β* = 0.50, *p* < 0.001), autonomy (*β* = 0.16, *p* < 0.01), and relatedness needs (*β* = 0.31, *p* < 0.001). Similarly, self-efficacy had a strong positive association with competence (*β* = 0.50, *p* < 0.001) and autonomy needs (*β* = 0.16, *p* < 0.001), while the path coefficient between self-efficacy and relatedness needs was not significant (*β* = 0.06, *p* = 0.121). There was a significant direct association between self-efficacy and exercise behavior (*β* = 0.17, *p* < 0.01). Next, the Bootstrap method analysis was used to test the mediating role of self-efficacy. The results indicated that self-efficacy statistically mediated the association between competence needs and exercise behavior (*β* = 0.07, *p* < 0.001, 95% CI [0.009–0.076]) and relatedness needs and exercise behavior (*β* = 0.05, *p* < 0.001, 95% CI [0.007–0.059]).

### 4.6 Moderation analysis

**Figure 2:**
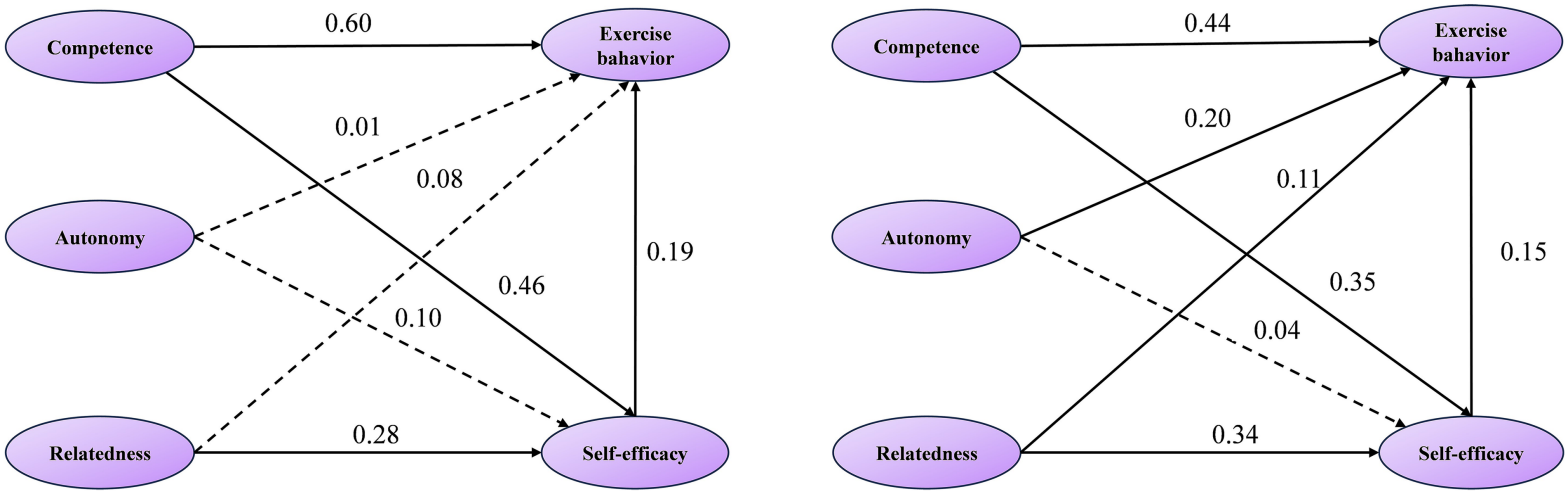
Structural equation model of basic psychological needs in PE, self-efficacy, and exercise behavior among different gender groups (left: Male students; right: female students).

To further investigate the psychological mechanism of basic psychological needs in PE on exercise behavior across gender groups, a multigroup analysis was conducted using AMOS. Before conducting the multi-group analysis, we conducted measurement invariance testing to investigate equivalence across gender. As shown in Table 3, the configural model (Model 1) had a good fit to the data across gender (*χ*^2^/df = 3.700, RMSEA = 0.042, GFI = 0.939, NFI = 0.929, IFI = 0.939, and CFI = 0.939). Fit indices of Model 1 suggested equivalent factor structure across gender and provided a baseline model to compare subsequent models. Next, we tested measurement weight invariance (Model 2) by constraining all factor loadings to be the same across gender. Changes in fit indices (ΔCFI ≤ 0.01 and ΔRMSEA ≤ 0.015) indicated that the Model 1 and Model 2 did not significantly differ. Finally, structural weight invariance (Model 3) was tested by constraining structural path coefficients among all latent variables to be equal across gender. The ΔCFI between Model 2 and Model 3 was 0.015 > 0.01, indicating that at least one structural path differed significantly in strength across gender. Therefore, we further performed Critical Ratio (C.R.) and path coefficient comparisons were conducted for each pathway to explore whether gender moderated the associations among variables within the mediation model. As shown in Table 4, the C.R. serves as the core indicator for judging the significance of cross-group path coefficient differences, with an absolute value of C.R. ≥ 1.96 indicating a statistically significant difference at the 0.05 level (Marici and Turliuc, 2012). Specifically, the path competence need → Exercise behavior showed a significant cross-group difference (C.R. = −3.128), with an effect size difference of 0.16. The path of autonomy need → Exercise behavior also exhibited a significant gender difference (C.R. = 2.647), and the effect size difference was −0.19. Additionally, the path competence need → self-efficacy showed a significant cross-group difference (C.R. = −2.083), with an effect size difference of 0.11. In contrast, the direct paths from relatedness need to exercise behavior, autonomy need to self-efficacy, relatedness need to self-efficacy, and self-efficacy to exercise behavior remained invariant across groups (Figure 2).

**Table 3:** Fit indices for multi-group invariance tests.

Test	*χ*^2^/df	GFI	NFI	IFI	CFI	RMSEA	ΔCFI	ΔRMSEA
Configural invariance (Model 1)	3.700	0.921	0.929	0.939	0.938	0.042		
Measurement weight invariance (Model 2)	3.540	0.920	0.928	0.938	0.937	0.045	−0.001	0.003
Structural weight invariance (Model 3)	3.530	0.920	0.928	0.927	0.922	0.047	−0.015	0.002

**Table 4:** Different path’ C.R. and the difference of effect size.

Path	Critical ratio (C.R.)	Effect sizes
Competence need → exercise behavior	−3.128	0.16
Autonomy need → exercise behavior	2.647	−0.19
Relatedness need → exercise behavior	−0.438	−0.03
Competence need → self-efficacy	−2.083	0.11
Autonomy need → self-efficacy	0.912	0.06
Relatedness need → self-efficacy	0.847	−0.06
Self-efficacy → exercise behavior	−1.262	0.04

## 5 Discussion

This study investigated the relationship between basic psychological needs in PE and exercise behavior among college students. It systematically explored the mediating role of self-efficacy and gender difference in the strength of the associations among the study variables, thereby providing new evidence for the application of SDT in the PE context. SEM results indicated that the needs for competence, autonomy, and relatedness all significantly positively associated with exercise behavior. Among these factors, competence need showed the strongest association with exercise behavior, followed by autonomy and relatedness needs, aligning with the findings of Zhang and Miao, 2024. The findings suggest that the satisfaction of basic psychological needs correlates closely with college students’ engagement in and maintenance of exercise behavior. The prior research supported the view that satisfaction of the competence need enhances individuals’ mastery of sports skills and confidence in tackling task challenges (Orkibi and Ronen, 2017), thereby forming a positive feedback loop of competence cognition-behavior reinforcement. Satisfaction of the autonomy need facilitates the internalization of motivation, which in turn improve students’ exercise engagement and persistence (Leo et al., 2022). Moreover, satisfaction of the relatedness need contributes to the development of positive social networks in sports contexts, strengthens individuals’ sense of belonging, and provides a psychological foundation for the adaptability and continuity of exercise behavior (Gucciardi and Jackson, 2015).

The results showed that the needs for competence and relatedness indirectly associated with college students’ exercise behavior through the mediating role of self-efficacy. This finding aligns with Bandura’s theory of self-efficacy and can be interpreted as follows: When individuals perceive improvements in their abilities, this heightened sense of competence strengthens the belief that “I am capable of completing” exercise tasks (Raven and Pels, 2021). Meanwhile, the emotional resources provided in PE such as teacher encouragement or peer support allow students to experience emotional satisfaction of being accepted and value in exercise settings. This emotional fulfillment is then internalized into the self-perception that “I am a valuable member of the team”, which reinforces the efficacy belief that “I can persist in group exercise” (Bandura, 1977). The synergistic interplay between these two needs manifests an interaction pattern of competence laying the foundation and relatedness providing empowerment, ultimately transforming these beliefs into sustained behavioral motivation for exercise participation.

SEM and multi-group analyses revealed gender-specific associational patterns in the relationships between basic psychological needs, self-efficacy, and exercise behavior, aligning with previous studies (Li et al., 2025; Weman-Josefsson et al., 2015). Grounded in SDT, these patterns reflect the contextual adaptability of basic psychological needs (Vansteenkiste and Ryan, 2013), potentially shaped by gender role socialization. For females, exercise behavior was associated with a multidimensional need pattern, in which competence, autonomy, and relatedness all showed significant associations. Although the associated strength of competence need to exercise behavior was weaker than that observed in males, competence remained the strongest predictor within the female group. This balanced pattern may reflect communal gender norms emphasizing autonomy support and social relatedness, consistent with SDT’s emphasis on person-context fit. In contrast, males exhibit a unidimensional, competence-focused pattern, with competence need showing the strongest association with exercise behavior, while autonomy and relatedness were less salient. This pattern aligns with masculinity norms emphasizing strength and competitiveness (Guo and Huang, 2025). Notably, some direct paths (e.g., relatedness need → exercise behavior; self-efficacy → exercise behavior) remained invariant across gender, indicating partially shared motivational processes. These findings suggest that gender role expectations may influence how psychological needs are prioritized, supporting SDT’s view that motivational processes are context-dependent. From an applied perspective, competence-oriented strategies may resonate more strongly with males, whereas females may benefit from interventions supporting competence, autonomy, and relatedness. However, these implications should be interpreted cautiously given the cross-sectional design.

Additionally, a gender difference was observed in the association between competence need and self-efficacy, with a stronger association observed in males. As this path demonstrated non-invariance, it represents a relatively robust gender-specific pattern. This finding aligns with social role theory, whereby competence fulfillment may more directly enhance males’ achievement motivation and self-efficacy (Deaner et al., 2016; Hentschel et al., 2019; Wang and Yu, 2023). In contrast, stereotype-related self-doubt may attenuate the translation of competence satisfaction into self-efficacy among females (Löffler and Greitemeyer, 2023). In particular, PE environments amplify this: teachers often encourage males to tackle challenges and provide reinforcing feedback (Pill and SueSee, 2017), while females face gendered pedagogical approaches prioritizing femininity over competence cultivation (Scraton, 2017). Overall, the results indicate that gendered socialization and contextual factors may shape how psychological needs are associated with self-efficacy and exercise behavior. Nevertheless, because the study is cross-sectional, the observed mediation reflects statistical associations rather than causal processes, warranting further longitudinal or experimental research.

However, this study had several limitations. First, we used the General Self-Efficacy Scale instead of a physical activity-specific self-efficacy instrument. Although this measurement is consistent with the study’s theoretical focus on general psychological resources and is widely used in adolescent health behavior research, it to some extent weakens the theoretical coherence with Social Cognitive Theory in the physical activity context and may reduce the measurement precision of the mediation analysis for physical activity behavior. Second, the study used a cross-sectional design, which cannot confirm causal relationships between basic psychological needs in PE, self-efficacy, and exercise behavior. The data only reflects a snapshot of variables at one time, failing to capture their dynamic evolutionary characteristics over time and leading to the psychological mechanism identified in this study being a correlational inference rather than a causal validation. Third, the study has unavoidable CMV risks: all variables were measured via self-reported questionnaires, and while procedural controls and a latent CMV factor method were used to reduce and detect CMV, these methods only partially mitigate such biases and cannot completely eliminate them, which may have led to an overestimation of the observed correlations among variables and the estimated strength of indirect associations. Finally, although participants were recruited from multiple universities across different provinces, with students inherently nested within these institutional clusters, institutional-level effects were not explicitly modeled in the analyses. This neglect of the clustered data structure may lead to underestimated standard errors and inflated Type I error rates, which could compromise the validity of the calculated standard errors and *p*-values due to potential intra-class correlation among students within the same university.

## 6 Conclusion

Promoting college students’ exercise behavior and enhancing their physical health constitute a core mission of PE in cultivating well-rounded talent at the university level. This study systematically explored the potential mechanism underlying the relationship between basic psychological needs in PE and college students’ exercise behavior. The results revealed significant group-specific distinctions and indicated that self-efficacy statistically mediated the associations between basic psychological needs and exercise behavior. Moreover, competence need showed a stronger association with self-efficacy among male students, suggesting that males tend to exhibit a relational pattern linked to self-efficacy gains through the process of improving their sports skills, which was in turn related to more persistent exercise behavior.

## Data Availability

The datasets presented in this study can be found in online repositories. The names of the repository/repositories and accession number(s) can be found in the article/Supplementary material.
